# PD212 A Model-Based Study To Estimate Medicare Payment Methods With Chinese Characteristics – The Diagnosis Intervention Packet Policy

**DOI:** 10.1017/S0266462324004331

**Published:** 2025-01-07

**Authors:** Chengxin Fan, Wenqiang Yin

## Abstract

**Introduction:**

The diagnosis intervention packet (DIP) is a health insurance payment method with Chinese characteristics. Because the DIP is easy to understand and operable, it has become the main payment method promoted by China’s National Health Insurance Bureau. The development and reform of the DIP policy, which was introduced in 2020, is worth evaluating.

**Methods:**

Policy texts were selected using conceptual sampling and expert interviews. The basic framework of policy evaluation was determined using text mining and statistical analysis methods. Word frequency analysis of the DIP payment policy content was undertaken using NVivo and Gephi software to compare the scope of concern before and after policy implementation. Quantitative evaluation of representative DIP health insurance payment policy content at the national level in China before and after policy implementation (2020 to 2022) was conducted by constructing a text mining and policy modeling consistency (TM-PMC) index model containing nine primary variables and 38 secondary variables.

**Results:**

Policy content analysis using text mining tools revealed that DIP-related policy themes were relatively concentrated, primarily focusing on disease types, medical institutions, and directories. These themes continue to be consistently updated. The quantitative results of the TM-PMC index model showed that the overall design of the policies was reasonable, but there was a noticeable variation in differentiation between the policies. Out of the eight policies analyzed, five were rated as excellent, two as good, and one as acceptable.

**Conclusions:**

The DIP policy is subject to continuous supplementation and optimization. The main factors that influence the value of the TM-PMC index for the policy include the following: policy objectives, policy objects (the groups for which the policy is implemented), policy tools, and policy perspectives. Therefore, various reforms related to collection and payment should be carried out in a locally adapted and standardized manner.

